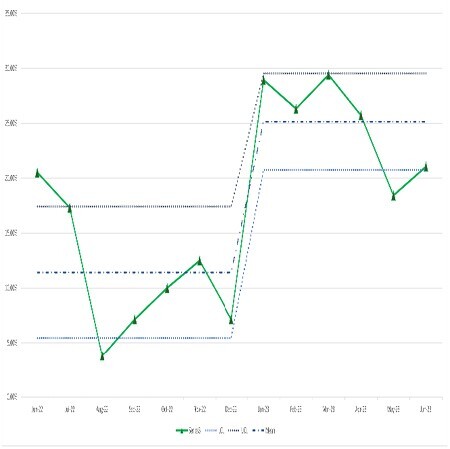# 540 Increasing Discharges Prior to 11am in Patients with Burn Injuries

**DOI:** 10.1093/jbcr/irae036.174

**Published:** 2024-04-17

**Authors:** Clinton D Leonard, Elizabeth L Slater, Denise Hargis, Richelle Graham, Maria Troche, Sabrina McGrew, Matthew Sigel, Anne L Wagner

**Affiliations:** Vanderbilt University, Nashville, TN; Vanderbilt University, Nashville, TN; Vanderbilt University, Nashville, TN; Vanderbilt University, Nashville, TN; Vanderbilt University, Nashville, TN; Vanderbilt University, Nashville, TN; Vanderbilt University, Nashville, TN; Vanderbilt University, Nashville, TN

## Abstract

**Introduction:**

This study describes a quality improvement project to increase the number of patient discharges prior to 11am in a high-volume Burn Center.

A limited supply of staffed inpatient beds creates capacity constraints and can adversely affect patient flow. Discharging patients earlier has been shown to promote throughput, improving length of stay (LOS), patient satisfaction, and decrease costs. Burn patients face unique barriers to discharge compared to other inpatient groups.

**Methods:**

This initiative utilized the Four Disciplines of Execution (4DX) framework, which focuses on identifying key drivers to achieve a specific, time-delimited quality improvement goal. Our primary target was increasing the number of discharges prior to 11am to 30% over the span of 6 months. Lead measures included (a) designation of a “priority discharge” patient, (b) discharge order entry prior to 0930, and (c) discharge prescriptions filled by the pharmacy the night before discharge. Baseline data was gathered from June 1st until Dec 11th 2022 and compared to the intervention period December 12th, 2022 through May 31st, 2023.

**Results:**

Discharges prior to 11am increased from 11.8% to 27.1% over the course of the study. Median discharge time improved by 72 minutes. Discharge order entry time improved by 121 minutes. Potential special-cause variation was identified in January 2023.

**Conclusions:**

Discharges prior to 11am were significantly increased after implementing a multidisciplinary approach between nursing, providers, case management and social work.

**Applicability of Research to Practice:**

The techniques we used to increase early discharges should be easily transferable to other Burn Centers, with positive impact on LOS and amount of time patients spend boarding in the ED.